# Preliminary Study on Rapid and Simultaneous Detection of Viable *Escherichia coli* O157:H7, *Staphylococcus aureu*s, and *Salmonella* by PMA-mPCR in Food

**DOI:** 10.3390/molecules28155835

**Published:** 2023-08-03

**Authors:** Yao Liu, Caijiao Wei, Hui Wan, Sarengaowa, Xiaoping Liang, Tao Jiang, Yuhe Dong, Xihong Zhao, Tian Zhong

**Affiliations:** 1 School of Pharmacy and Food Science, Zhuhai College of Science and Technology, Zhuhai 519041, China; liuyao@zcst.edu.cn (Y.L.); sarengaowa@zcst.edu.cn (S.); c15875684354@hotmail.com (X.L.); jiangtao@zcst.edu.cn (T.J.); 2 School of Environmental Ecology and Biological Engineering, Wuhan Institute of Technology, Wuhan 430205, China; angelcjwei@foxmail.com; 3 Nanchang Agricultural Technology Popularization Center, Nanchang 330299, China; 18970804331@163.com; 4 Faculty of Medicine, Macau University of Science and Technology, Taipa, Macao 999078, China; dongyuheff@foxmail.com

**Keywords:** foodborne pathogens, propidium monoazide (PMA), multiplex PCR, viable cell, detection method

## Abstract

*Escherichia coli* O157:H7, *Staphylococcus aureus*, and *Salmonella* are major foodborne pathogens that are widespread in nature and responsible for several outbreaks of food safety accidents. Thus, a rapid and practical technique (PMA-mPCR) was developed for the simultaneous detection of viable *E. coli* O157:H7, *S. aureus*, and *Salmonella* in pure culture and in a food matrix. To eliminate false positive results, propidium monoazide (PMA) was applied to selectively suppress the DNA amplification of dead cells. The results showed the optimum concentration of PMA is 5.0 µg/mL. The detection limit of this assay by mPCR was 10^3^ CFU/mL in the culture broth, and by PMA-mPCR was 10^4^ CFU/mL both in pure culture and a food matrix (milk and ground beef). In addition, the detection of mixed viable and dead cells was also explored in this study. The detection sensitivity ratio of viable and dead counts was less than 1:10. Therefore, the PMA-mPCR assay proposed here might provide an efficient detection tool for the simultaneous detection of viable *E. coli* O157:H7, *S. aureus*, and *Salmonella* and also have great potential for the detection and concentration assessment of VBNC cells.

## 1. Introduction

In recent years, with the frequent emergence of various types of food safety incidents, foodborne pathogens have increased food safety risks and posed a great threat to people’s health and public safety [1]. *Escherichia coli* O157:H7, *Staphylococcus aureus*, and *Salmonella* are considered to be the three most important foodborne pathogens, which widely exist in many kinds of foods and are frequently reported as agents causing food poisoning [2,3]. *E. coli* O157: H7 is the most well-known and most studied serotype, which can cause gastroenteric infections and lead to serious kidney damage and even death at a low infectious dose. According to other studies, *S. aureus* is also the cause of frequent food poisoning. In 2017, an estimated 119,247 *S. aureus* bloodstream infections with 19,832 associated deaths occurred in the United States [4]. Among foodborne disease outbreaks, *Salmonella* is ubiquitous in a wide range of foods and is the second most commonly reported foodborne pathogen, and leads to bacterial gastroenteritis [5,6,7]. CDC estimates *Salmonella* bacteria cause about 1.35 million infections, 26,500 hospitalizations, and 420 deaths in the United States every year [8]. However, dead pathogenic bacteria will lose their pathogenicity, but the intact DNA of dead cells can persist for a considerable time and easily lead to a high incidence of false-positive results. Therefore, the detection of the presence and content of viable pathogens is the focus of attention for the management and monitoring of food safety. 

The traditional detection methods of foodborne pathogens rely on microbial isolation, culture, and biochemical identification technology. However, culture-based methods are so time-consuming and laborious that it is difficult to meet the new challenges of food companies and the demands for fast and accurate detection of foodborne pathogens by regulatory authorities [9]. Moreover, conventional methods also easily fail to detect viable but non-culturable (VBNC) cells that are pathogenic but non-colony forming. Hence, it is urgent to establish a rapid and accurate method for the simultaneous detection of foodborne pathogens.

To date, polymerase chain reaction (PCR) has become the most widely used technology in classical microbiological methods because of its advantages of short analysis time, strong specificity, and a high degree of automation [10]. Compared with ordinary PCR, multiplex polymerase chain reaction (mPCR) can amplify multiple fragments in a single reaction system for the simultaneous detection of multiple pathogenic bacteria, which has more advantages in saving time, laboratory costs, and reagents [11]. However, normal mPCR is unable to differentiate viable cells from dead cells because the DNA from dead bacteria has a certain persistence, which can also serve as a template during the PCR reaction and lead to a high incidence of false-positive results [12,13]. Thus, this is a major challenge for detecting viable cells in mixed samples of dead and viable microbial cells. In order to overcome this drawback, nucleic acid intercalating dyes, such as ethidium monoazide (EMA) or propidium monoazide (PMA), has been used as sample pre-treatment to remove the interference of dead cells [14,15]. PMA (or EMA) is a high-affinity dye for DNA that cannot enter through intact cell membranes but can selectively permeate into membrane-compromised or dead cells and form cross-links with DNA through exposure to strong visible light. This modification can inhibit PCR amplification to eliminate positive signals from dead bacteria [16,17]. However, it has been reported that EMA has higher toxicity to viable cells than PMA and could partly penetrate into the membranes of viable cells [18,19,20,21]. Therefore, in this study, PMA was selected as pretreatment in combination with mPCR for the fast and simultaneous detection of viable *E. coli* O157:H7, *S. aureus*, and *Salmonella.* To achieve the objective of this study, we selected the O157 antigen gene (*rfb*E) of *E. coli* O157:H7, the thermostable nuclease gene (*nuc*) of *S. aureus*, and an invasive gene (*inv*A) of *Salmonella* as highly specific and sensitive primers to detect the three foodborne pathogens. Meanwhile, the PMA-mPCR assay was evaluated in artificially contaminated food products. 

## 2. Results

### 2.1. Optimization of Concentration of PMA Treatment

**Figure 1:**
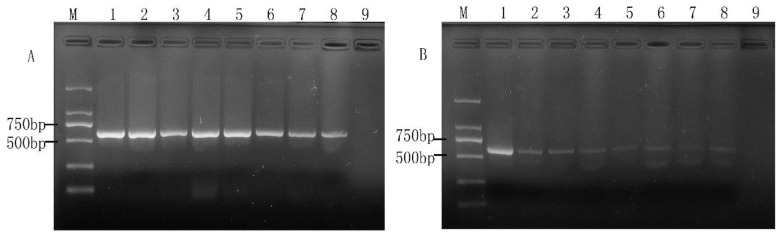
Concentrations of PMA for inhibiting the amplification of DNA from 10^8^ CFU/mL of *Escherichia coli* O157: H7 viable cells (**A**) and dead cells (**B**). (**A**) Lanes 1~8, varying concentrations of PMA (0, 1, 2, 3, 5, 10, 15, 20 µg/mL, lane 9, negative control. M, DL 2000 DNA Marker; (**B**) Lanes 1~8, varying concentrations of PMA (0, 1, 2, 3, 5, 7, 10,15 µg/mL), lane 9, negative control.

**Figure 2:**
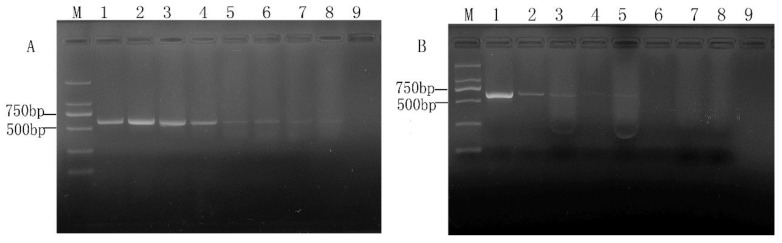
Concentrations of PMA for inhibiting the amplification of DNA from10^6^ CFU/mL of *Escherichia coli* O157: H7 viable cells (**A**) and dead cells (**B**). (**A**) Lanes 1~8, varying concentrations of PMA (0, 1, 2, 3, 5, 10, 15, 20 µg/mL, lane 9, negative control. M, DL 2000 DNA Marker; (**B**) Lanes 1~8, varying concentrations of PMA (0, 1, 2, 3, 5, 7, 10, 15 µg/mL), lane 9, negative control.

**Figure 3:**
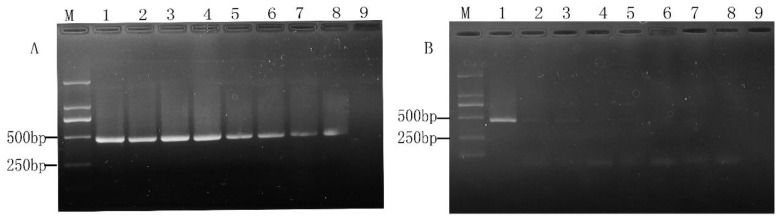
Concentrations of PMA for inhibiting the amplification of DNA from10^6^ CFU/mL of *Staphylococcus aureus* viable cells (**A**) and dead cells (**B**). (**A**) Lanes 1~8, varying concentrations of PMA (0, 1, 2, 3, 5, 10, 15, 20 µg/mL, lane 9, negative control. M, DL 2000 DNA Marker; (**B**) Lanes 1~8, varying concentrations of PMA (0, 1, 2, 3, 5, 7, 10, 15 µg/mL), lane 9, negative control.

**Figure 4:**
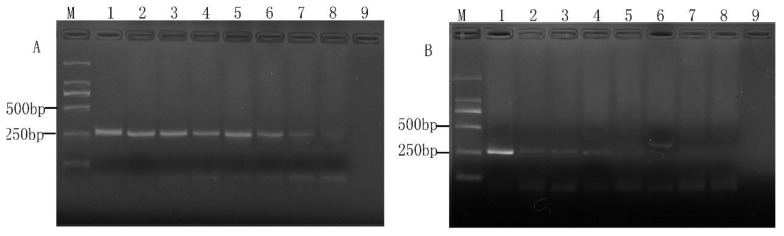
Concentrations of PMA for inhibiting the amplification of DNA from10^6^ CFU/mL of *Salmonella* viable cells (**A**) and dead cells (**B**). (**A**) Lanes 1~8, varying concentrations of PMA (0, 1, 2, 3, 5, 10, 15, 20 µg/mL, lane 9, negative control. M, DL 2000 DNA Marker; (**B**) Lanes 1~8, varying concentrations of PMA (0, 1, 2, 3, 5, 7, 10, 15 µg/mL), lane 9, negative control.

PMA concentration is an important factor in the detection of viable target bacteria. Because there are some differences between cell membranes and cell walls of different microorganisms, the optimal PMA concentration may also be different. Therefore, a series of PMA concentrations were conducted on the three viable and dead cell samples in this study. For *E. coli* O157: H7, two concentration gradients (10^8^ CFU/mL, 10^6^ CFU/mL) were explored. The results are shown in Figure 1 and Figure 2, respectively. It can be seen from Figure 1A that PMA had no significant inhibition on viable *E. coli* O157:H7 cells. Whereas for the 10^8^ CFU/mL of dead cells, the PMA amplification of its target band appeared significantly weakened but did not completely disappear (Figure 1B). It is indicated that the concentration of dead cells may exceed the maximum range of DNA molecules with which PMA can be combined. When *E. coli* O157:H7 cells were about 10^6^ CFU/mL, PMA had a slight inhibitory effect on viable cells after the PMA concentration reached 10 µg/mL (Figure 2A). Therefore, the PMA concentration range for cells could not be over 10 µg/mL. As for the concentration of dead bacteria, the amplification of DNA was completely inhibited when PMA at a concentration of 5.0 µg/mL or higher was applied (Figure 2B). According to the study of PMA on the impact of *E. coli* O157:H7, we directly explored the effect of PMA on the viable and dead bacteria of 10^6^ CFU/mL *S. aureus* and *Salmonella.* All the results can be seen in Figure 3 and Figure 4; there was no obvious difference between the amplification of target DNA derived from viable cells of *S. aureus* and *Salmonella* treated with a series of PMA concentrations. For the dead *S. aureus* and *Salmonella* cells, when the concentration of PMA was (or was over) 3.0 and 5.0 µg/mL, respectively, the amplification of DNA can be completely inhibited (Figure 3B and Figure 4B). Hence, the optimum concentration of PMA for distinguishing those target bacteria DNA from dead and viable cells by the PMA-mPCR is 5.0 µg/mL.

### 2.2. Specificity and Sensitivity of PMA-mPCR in Pure Culture

**Figure 5:**
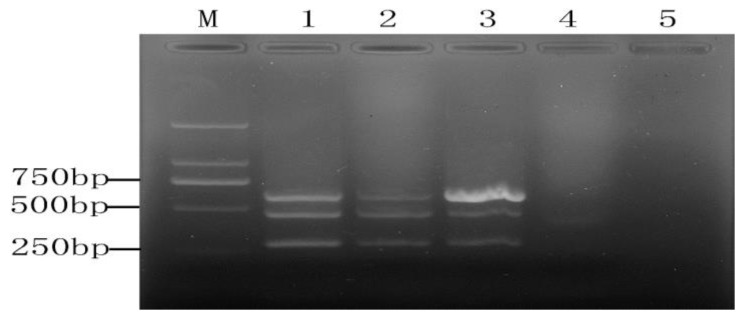
Specificity test of PMA-mPCR. M: DL 2000 DNA Marker; lane 1, viable mixture (containing 10^6^ CFU/mL viable *Listeria ivanovii* subsp.) without PMA, lane 2, with PMA viable mixture (containing 10^6^ CFU/mL viable *Listeria ivanovii* subsp.), lane 3, dead bacteria mixture (containing 10^6^ CFU/mL of dead *Listeria ivanovii* subsp.) without PMA, lane 4, dead bacteria mixture (containing 10^6^ CFU/mL dead *Listeria ivanovii* subsp.) with PMA, lane 5, negative control.

To investigate whether the presence of non-target bacteria will interfere with the detection of the three target bacteria, non-target bacteria (*Listeria ivanovii* subsp. about 10^6^ CFU/mL) was mixed with 10^6^ CFU/mL of the three target bacteria to determine the specificity of the PMA-m PCR assay. The results presented in Figure 5 indicate that non-target bacteria did not disturb the identification and detection of the target bacteria, and there was also no cross-reactivity among the different strains. Moreover, all the amplified fragments were consistent with our expectations. For instance, the amplified fragment of *E. coli* O157:H7 was 601 bp, of *S. aureus* near 500 bp was 484 bp, and of *Salmonella* within 250–500 bp was 284 bp. It also presented that each primer set was highly species-specific.

**Figure 6:**
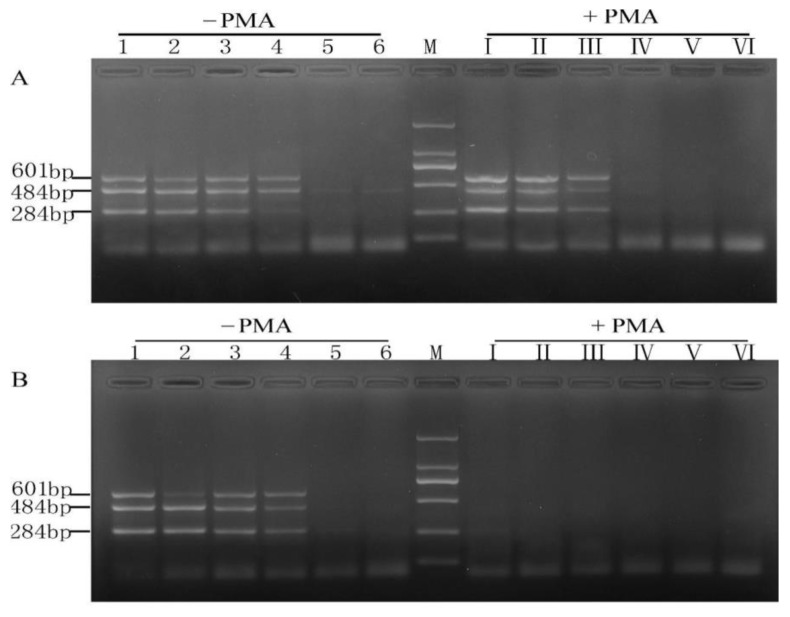
The limit of detection of multiplex PCR assay using 10-fold serially diluted viable target cells(**A**) and dead target cells (**B**) with (+) and without (−) PMA treatment. M, DL 2000 DNA Marker; lanes 1~6 (or Ⅰ~Ⅵ), varying concentrations of bacterial suspension (10^6^, 10^5^, 10^4^, 10^3^, 10^2^, and 10^1^ CFU/mL).

In order to explore whether the detection limits of mPCR were affected by PMA treatment, all the ten-fold serial dilutions of the three mixed target bacterial cultures were divided into two groups in this study. One group was subjected to PMA treatment, while the other was a control. Without PMA treatment of viable and dead bacteria, the detection limits in pure culture were 10^3^ CFU/mL (4.2 × 10^3^ CFU/mL for *E. coli* O157:H7, 2.1 × 10^3^ CFU/mL for *S. aureus*, 5.4 × 10^3^ CFU/mL for *Salmonella*), as shown in Figure 6A,B. The detection limit of the viable bacteria treated by PMA was 10^4^ CFU/mL, and all the dead bacteria treated by PMA had no signals. This result was consistent with that reported by Zhang et al. [12] and also indicated that PMA treatment of cells was efficient and essential before DNA extraction.

### 2.3. Sensitivity of the PMA-mPCR Assay in Artificial Contaminated Food Products

**Figure 7:**
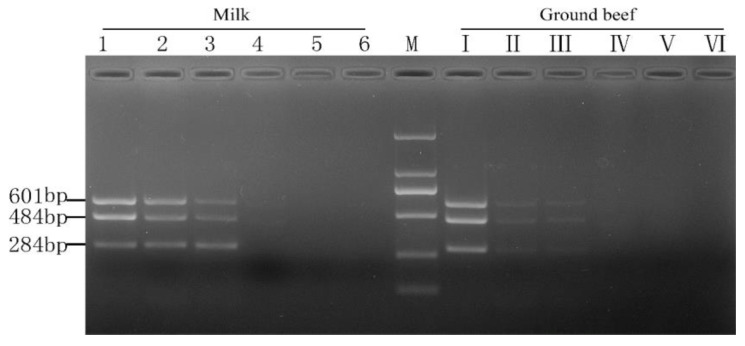
The limit of detection of PMA-mPCR in the detection of viable target bacteria in milk and ground beef. M, DL 2000 DNA Marker; lanes 1~6 (or Ⅰ~Ⅵ), varying concentrations of bacterial suspension (10^6^, 10^5^, 10^4^, 10^3^, 10^2^, and 10^1^ CFU/mL).

The efficiency of PMA-mPCR was further confirmed by using artificially contaminated aseptic deluxe pure milk and ground beef with concentrations of three mixed viable target bacterial cultures ranging from 10^1^ to 10^6^ CFU/mL to demonstrate real sample application. Figure 7 shows that the detection limits of *E. coli* O157:H7, *S. aureus*, and *Salmonella* in both food matrices all were 10^4^ CFU/mL. The composition of aseptic deluxe pure milk and ground beef was more complex than that of the pure culture system, but the detection limits of three viable target bacteria were consistent with the pure medium utilized in this experiment. The result confirmed that the PMA-mPCR assay could effectively detect three viable target bacteria in food samples.

### 2.4. Detection of Mixed Viable and Dead Cells

**Figure 8:**
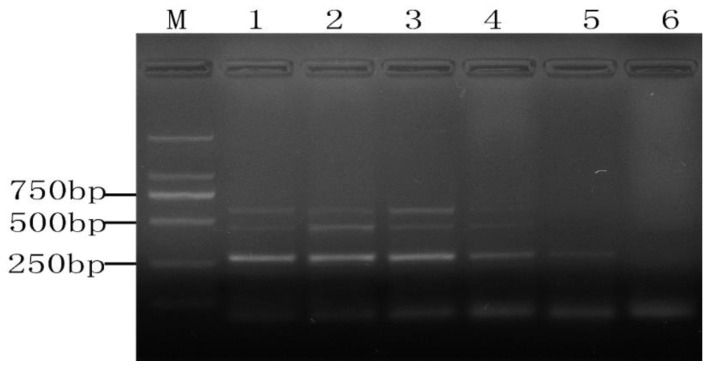
The results of PMA-mPCR in the mixture of viable and dead cells. M, DL 2000 DNA Marker; lanes 1~6, viable bacteria accounted for the proportion of dead bacteria (10^6^ CFU/mL) were 1: 1, 1: 2, 1: 5, 1:10, 1: 100, 1:1000.

The bacteria present in food do not just exist in a viable or dead form; some of them exist in the form of a mixture of viable and dead cells. Moreover, according to some researchers’ previous studies on viable but non-culturable bacteria, many bacterial cells entering the VBNC state might also be in the same mixed state [22,23]. Hence, the detection of mixed viable and dead cells was carried out in this study. As can be seen from Figure 8, when the proportion of viable and dead bacteria (10^6^ CFU/mL) was lower than 1:10, there was no signal found by PMA-mPCR conducted at a concentration of 5 μg/mL. It indicated that the limit of detection of the three target viable cells was about 10^5^ CFU/mL in the mixture of viable and dead cells culture. Additionally, it also suggests that the amplification of DNA from both dead and viable cells may be simultaneously inhibited when the ratio of viable and dead cell counts is less than 1:10. Because the difference between the final concentration of viable cells in the mixture of viable and dead cells culture and the single viable cells is not so great, the viable amplification is a certain correlation between the ratio of viable: dead cells. Liu and Mustapha also reported that the low concentrations (10^4^–10^0^ cells/g) of viable and 10^6^ dead cells/g contaminating the ground beef sample also generated negative results using PMA real-time PCR [14]. Furthermore, Zhong et al. [24] also got similar results.

## 3. Materials and Methods

### 3.1. Bacterial Strains and Cultivation

Reference strains of *Salmonella* (ATCC 13076), *S. aureus* (ATCC 27664), and *E. coli* O157:H7 (ATCC 43895) were used to establish the PMA-mPCR assay in this study. *Listeria ivanovii* subsp (ATCC 19119) was used as a negative control. All bacterial strains were inoculated on Difco^™^ Tryptic Soy Agar (TSA) at 37 °C for 24 h and then cultured in Bacto^™^ Tryptic Soy Broth (TSB) at 37 °C in a rotary shaker at 190 rpm for 12 h. After incubation at 37 °C for 24 h, the bacterial count was determined by the plate counting dilution method.

### 3.2. Preparation of Viable and Dead Cells

To obtain fresh bacterial suspensions, the strains were cultured in Bacto^™^ Tryptic Soy Broth in a rotary shaker at 190 rpm for 12 h, and the plates were incubated at 37 °C for 24 h before enumeration. Then, 1 mL of overnight culture of fresh cells suspension (about 10^8^ CFU/mL) was transferred into a 1.5 mL microcentrifuge tube and diluted with sterile normal saline to make a bacterial concentration of 10^6^ CFU/mL through serial decimal dilutions. Following this, all the bacterial suspensions were centrifuged at 10,000× *g* for 5 min, washed three times, and then resuspended in equal volumes of physiological saline as the displaced TSB. In order to get dead cells, all the cell suspension was killed by exposure to 100 °C for 5 min in a water bath. Then the treated bacterial suspension was cooled to room temperature. The method for confirming dead cells refers to the literature [2,6,14], performing the plate count method on TSA and culturing in TSB after incubation at 37 °C for 24 h. Meanwhile, three parallel experiments were performed to ensure all the cells had died. 

### 3.3. PMA Treatment and DNA Extraction

PMA (Biotium, Inc., Hayward, CA, USA) was dissolved in 20% (*v/v*) dimethyl sulfoxide (DMSO) (Hayashi Pure Chemical Industries, Ltd., Oakville, Osaka, Japan) to obtain a PMA stock solution (5.0 mg/mL) and stored at 4 °C in the dark. To obtain a PMA working solution with a final concentration of 0.5 mg/mL, the PMA stock solution needed to be diluted 10-fold before it was used. The PMA treatment method was slightly modified according to the procedures described in previous research [24,25]. Briefly, 500 μL of the prepared bacterial suspension in a light-transparent 1.5 mL sterile centrifuge tube was mixed with a series of concentrations of PMA solution and then stored on ice in the dark for 10 min. This promoted PMA entering the dead cells and intercalating with the DNA. After incubation in the dark, all the sample mixtures were placed horizontally on ice and were exposed to a 650 W halogen lamp (Osram, Germany) for 5 min at a 20 cm distance from the light source. During exposure, in order to guarantee homogeneous light exposure, all the tubes were shaken every 30 s. After the light exposure, all the PMA-treated cells were centrifuged at 1000× *g* for 5 min and washed three times with sterilized saline water. 

DNA extraction was based on thermal lysis according to the protocol described in a previous study [25]. Briefly, the PMA-treated pelleted cells (viable or dead) were prepared for DNA extraction. The cells were resuspended with 500 μL Tris–EDTA Triton buffer solution and were boiled in a water bath for 10 min, followed by immediate immersion in ice for 10 min. Then, the samples were centrifuged at 10,000× *g* for 10 min, and the supernatant was used as a DNA template.

### 3.4. Multiplex PCR Conditions

In our previous study, when the annealing temperature was at 57 °C and *rfb*E primer, *nuc* primer, and *inv*A primer were 0.1, 0.2, and 0.4 μM respectively, we obtained the most specific and sensitive amplification bands for the three target genes [26]. The primer sequences are listed in Table 1. The primers were synthesized and purified by Tianyi Biotech (Wuhan, China).

The mPCR reaction was carried out in a 25 μL volume of the reaction mixture, which contained 0.5 μL of TaKaRa Taq^TM^ DNA polymerase (5 U/μL), 2.0 μL of dNTP Mixture (2.5 mmoL/L) (TaKaRa Biotech, Dalian, China), 2.5 μL of TaKaRa Taq^TM^ 10 × PCR Buffer (Mg^2+^ plus), 3 μL of DNA templates, 0.1 μM of each forward and reverse *rfb*E primer, 0.2 μM of each forward and reverse *nuc* primer, and 0.4 μM of each forward and reverse *inv*A primer. Then, ultra-pure water was filled up to a final volume of 25 μL. The mPCR conditions were as follows: pre-incubation at 94 °C for 5 min, followed by 30 cycles of denaturation at 94 °C for 30 s, annealing at 57 °C for 30 s, and elongation at 72 °C for 30 s, with a final extension at 72 °C for 10 min in a Life Pro Thermal Cycler (Hangzhou Bioer Technology Co. Ltd., Hangzhou, China). After the reaction was completed, the PCR amplification products were subjected to 2% agarose gel electrophoresis and visualized with Golden View dye by the BD-3000 Gel Image Analysis System (Beijing QHBODA Technology Co., Ltd., Beijing, China).

### 3.5. Specificity and Sensitivity of the PMA-mPCR

First, we prepared a mixed solution of the three target bacteria. We took 100 μL each of *Escherichia coli* O157: H7, *Staphylococcus aureus*, and *Salmonella bacteria* with a concentration of 10^7^ CFU/mL-10^2^ CFU/mL, respectively. They were added to 700 μL of normal saline one after another, stirred well, and a mixed solution with a target bacterial concentration of 10^6^ CFU/mL–10^1^ CFU/mL was obtained. To verify the specificity of target primers and investigate whether the presence of non-target bacteria will disturb the identification and the simultaneous detection of the three target bacteria, non-target cells *Listeria ivanovii subsp* (viable or dead) were tested by PMA-mPCR assay. At the same time, the negative controls and samples without PMA treatment in mPCR reaction were also subjected to evaluation. Additionally, in order to further test the limit of detection (LOD) of the PMA-mPCR assay, according to Shekar, A. et al. described in [2], tenfold serially diluted target bacteria (viable or dead) ranging from 10^6^ to 10^1^ CFU/mL were washed three times and resuspended in sterilized saline water. The suspensions were subjected to PMA treatment with the best-optimized concentration, and DNA was extracted using the above-mentioned method, followed by the mPCR assay. Furthermore, to explore whether the PMA treatment would have an effect on the sensitivity, non-PMA treated, and negative control samples were also subjected to the mPCR assay.

### 3.6. Detection of Viable Target Pathogens in Artificially Contaminated Food Products

Samples were treated according to Li et al. described previously with a few modifications [28]. Ground beef (Yayouwang Food Co., Ltd., Shantou, China) and aseptic deluxe pure milk (Inner Mongolia Mengniu Dairy Co., Ltd., Hohhot, China) were used for artificially contaminated studies. First, 5 g of ground beef was added to 45 mL of LB to obtain the sample mixture and homogenized in a mortar to produce a 1:10 homogenate. Then, we took another 7.0 mL of the two food homogenates and added 1.0 mL of the *E. coli* O157: H7, *Staphylococcus aureus*, and *Salmonella bacteria* cultures with a concentration ranging from 10^7^ CFU/mL to 10^2^ CFU/mL, respectively, and shook them well to obtain two food samples with three target bacterial concentrations of 10^6^ CFU/mL to 10^1^ CFU/mL. When all the samples (ground beef, aseptic deluxe pure milk) were verified negative for 3 target pathogens by traditional culture method and standard PCR, these samples were used for further artificial contamination experiments. Afterward, both food samples received the target bacteria, and they were uniformly mixed by shaking to obtain 3 target bacterial concentrations ranging from 10^1^ CFU/mL to 10^6^ CFU/mL, respectively. Subsequently, all samples were subjected to DNA extraction and mPCR sensitivity tests, as mentioned above. All of the tests were conducted in triplicate.

### 3.7. Detection of Mixed Viable and Dead Cells

**Figure 9:**
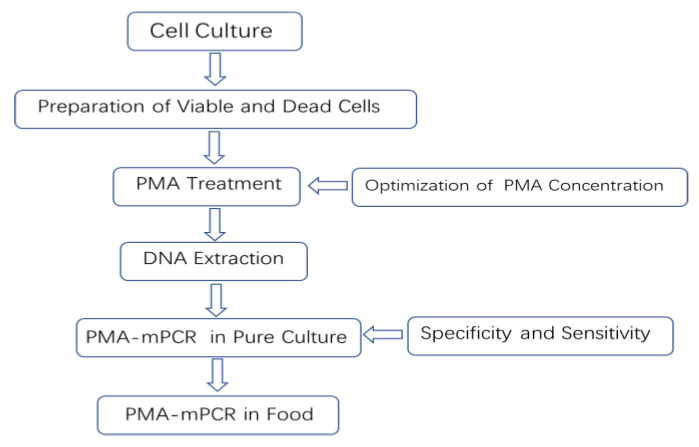
Schematic diagram of the PMA-mPCR in food in this study.

To evaluate whether the PMA-PCR method has an effect on the detection of mixed viable and dead cells, all dead target bacteria (about 10^6^ CFU/mL) were mixed with a series of corresponding viable target bacteria to obtain different proportions (1:1, 1:2, 1:5, 1:10, 1:100, and 1:1000) of viable and dead bacteria mixture suspensions. Thereafter, all the different ratios of viable and dead bacteria mixture suspensions were treated with an optimized concentration of PMA and then used for the PMA-mPCR assay. In this study, we sought to establish a simple method of rapid detection of viable *Escherichia coli* O157:H7, *Staphylococcus aureus*, and *Salmonella* by PMA-mPCR in food, as shown in Figure 9.

## 4. Discussion and Conclusions

According to previous reports, *E. coli* O157:H7, *S. aureus*, and *Salmonella* are common causative agents of food poisoning outbreaks, resulting in major public health issues and substantial economic burdens [29]. Although some methods for the detection of foodborne bacteria have been reported, many of them could not discriminate viable cells from dead cells. Therefore, it is urgent to establish a rapid and accurate method to detect these viable foodborne pathogens.

In recent years, melt curve-based real-time quantitative PCR assays have emerged as powerful tools for the detection of various pathogens, but it also has a disadvantage in that it requires expensive probes and fluorescent dye for the detection of various bacteria and will greatly increase the cost of the experiment. In addition, there is also a major challenge in detecting viable cells in mixed samples of viable and dead microbial cells by ordinary real-time quantitative PCR. Owing to the convenience and rapid characteristics of mPCR and being cheaper than real-time PCR [30,31], we investigated and established a PMA-mPCR assay that could simultaneously detect viable *E. coli* O157:H7, *S. aureus*, and *Salmonella*. Considering that PMA treatment is a key step to eliminate the false-positive results from dead cells, we therefore explored the optimal concentration of PMA from 0 to 20 µg/mL. In our study, the results show that PMA has less suppression for 10^8^ CFU/mL of dead *E. coli* O157:H7 cells, even though the PMA concentration is 15 µg/mL. Nevertheless, when PMA is at 5 µg/mL concentration, the results suggest that it can effectively inhibit the dead cell signals from 10^6^ CFU/mL of *E. coli* O157:H7, *S. aureus*, and *Salmonella*. At the same time, there is no difference between with or without PMA treatment on those three viable target cells, which is in accordance with previous studies by Yang et al. [6] and Forghani et al. [32]. However, the concentration of PMA (5 µg/mL) used in our study is low, which also can reduce the cost per assay compared to other reported studies [2,11]. Based on the optimized PMA concentration, the PMA-mPCR assay was successfully subjected to detect *E. coli* O157:H7, *S. aureus*, and *Salmonella* in both pure culture and artificial contaminated model food systems. The concentration of PMA depends on the number of background dead cells. Therefore, when the dosage of PMA is determined, the appropriate concentration of the dead bacteria does not cause false-positive results. If the concentration of dead cells exceeds the appropriate range, false positive results will occur. In this study, a PMA-mPCR assay was developed to rapidly detect the DNA from viable target bacteria in food samples, which cannot detect RNA directly. The total time required for analysis is about 4–6 h, including sample preparation. The detection sensitivity for those three pathogenic bacteria by mPCR is 10^3^ CFU/mL in pure culture, whereas it is 10^4^ CFU/mL in both pure culture and model food systems by PMA-mPCR. These detection sensitivity levels are consistent with our previous studies by mPCR [26] and similar to Zhong et al. [9]. The sensitivity of this study is not very high. This result can be explained by the fact that the PMA may also have little suppression for viable cells, or the thermal lysis method to extract DNA is not efficient. Nevertheless, the thermal lysis method could save time and eliminate the need for intensive labor [12], and the sensitivity of PMA-mPCR in our assay is similar to the result of Li et al. using a DNA extraction kit [28]. They also indicated that the PMA-mPCR detection system could effectively eliminate the signals from dead bacteria and has good stability and sensitivity in both pure culture and model food systems. When conducting foodborne pathogen detection in actual food samples, the difficulty of the pre-enrichment step mainly arises from the competition of other microorganisms present in the samples, the complexity of the food samples, and the low enrichment efficiency. To address these challenges, the following approaches can be adopted: optimizing the enrichment medium, such as using the method of immunomagnetic bead adsorption [6], optimizing the enrichment conditions [11,33], and using automated enrichment equipment based on the same principle of immunomagnetic bead adsorption. For rapid detection of foodborne pathogens, Kim’s group recently developed an HRPzyme-integrated PCR colorimetric detection platform and a paper chip device-based recombinase polymerase amplification method, which provided simple, fast, cost-effective and user-friendly detection assays for DNA as a target analyte [34,35,36]. The PMA-mPCR developed in this study may be expanded for quantitative analysis or semi-quantitative analysis, if needed, by combining with the application of ImageJ in colorimetric image data acquisition.

Viable but non-culturable (VBNC) foodborne pathogens are characterized by a loss of culture ability on enriched agar media and exhibit detectable metabolic functions, which may retain their ability to express toxic genes [37]. VBNC cells can easily fail detection and pose a risk to public health. Thus, many investigators have conducted VBNC induction studies on pathogenic bacteria. For instance, Zhao et al. [22] reported that VBNC cell counts still approximated 10^6^ cells/mL while the total cell counts of 10^8^ cells/mL declined to below 0.1 CFU/mL to undetectable levels. It indicates that the VBNC state is a mixture of viable and dead cells. Hence, we used the PMA-mPCR to detect a series of different proportions of the mixture of viable and dead cells. Meanwhile, we obtained that the detection limit of this assay to the viable cells mixed with dead cells (10^6^ CFU/mL) was 10^5^ CFU/mL. Namely, this method can be applied to the detection of VBNC cells at concentrations 10^5^ CFU/mL or higher. Although the method has some shortcomings and the detection limit is not low, it will have great potential for the detection and concentration assessment of VBNC cells.

In conclusion, a sensitive, specific, and convenient PMA-mPCR assay was developed to simultaneously detect viable *E. coli* O157:H7, *S. aureus*, and *Salmonella*, which was also applied to complex food matrices. The results of the PMA-mPCR method were consistent with traditional mPCR assay. This PMA-mPCR method might provide an efficient detection tool for monitoring food contaminants from these three pathogens to reduce the potential hazards of these harmful pathogens. Thus, it is anticipated that the method can be a sensitive, accurate, cost-effective, and potential tool for the rapid identification of multiple viable foodborne pathogens.

## Figures and Tables

**Table 1:** Primers used in this study.

Microorganism	Target Gene	Primer Sequence (5′-3′)	G + C (%)	Tm (°C)	Amplicon Length (bp)	Reference
*E. coli* O157:H7	*rfb*E (S83460)	F:GCCACCCCCATTTTCGTTG R:TCCTCTCTTTCCTCTGCGGT	57.9 47.4	63.2 51.7	601	[26]
*S. aureus*	*nuc* (AP017922)	F:TACAGGTGACTGCGGGCTTATC R:CTTACCGGGCAATACACTCACTA	50 45.4	60.2 58.3	484	[27]
*Salmonella*	*inv*A (M90846)	F:CTTTAGCCAAGCCTTGACGAAC R:AAAGGCAATACGCAAAGAGGT	54.5 47.8	62.1 60.6	284	[27]

## Data Availability

Data are available from the corresponding author.

## Sample Availability

Not available.
